# The Cyclopædia of Practical Medicine

**Published:** 1845-01

**Authors:** 


*The Cyclopædia of Practical Medicine.* Edited by John Forbes, M. D., Alexander Tweedie, M. D., and John Conolly, M. D. *Revised, with additions*, by Robley Dunglison, M. D.

Part XX. of this valuable work, which is now before us, treats of the following subjects, viz.: Scrofula, by Dr. Cumin; Sedatives, by Drs. Thomson and Dunglison; Sex, Doubtful, Dr. Beatty; Small-pox, Dr. Gregory; Softening of Organs, Dr. Carswell; Somnambulism and Animal Magnetism, Dr. Prichard; Spermatorrhœa, Dr. Dunglison; Spinal Marrow, Diseases of, Dr. Todd; Spleen, Diseases of, Drs. Bigsby and Dunglison; Statistics, Medical, Drs. Hawkins and Dunglison; Stethoscope Dr. Williams; Stimulants, Dr. A. T. Thomson; Stomach, Organic Diseases of, Dr. Houghton.

It will be seen, from the table of contents, that this important work continues to embrace subjects of the greatest interest; and from the progress made in its publication, it will be perceived that it advances from number to number with the regularity of a daily newspaper. Only *four parts* remain for its completion, and these, there can now be no doubt, will be forthcoming with the same regularity as those which have preceded; so that, in a few weeks, we shall be in possession of the entire work.

